# Toward a Molecular Understanding of Adaptive Immunity: A Chronology, Part III

**DOI:** 10.3389/fimmu.2014.00029

**Published:** 2014-02-04

**Authors:** Kendall A. Smith

**Affiliations:** ^1^Department of Medicine, Division of Immunology, Weill Medical College, Cornell University, New York, NY, USA

**Keywords:** adaptive immunity, TCR signaling, cell-mediated cytolysis, perforin, granzymes, IL-4-6, CD40, CD40-ligand

## Abstract

Early reports on T cell antigen receptor (TCR) signaling uncovered a rapid increase in intracellular calcium concentration and the activation of calcium-dependent protein kinase as necessary for T cell activation. Cytolytic T cell clones were instrumental in the discovery of intracellular cytolytic granules, and the isolation of the perforin and granzyme molecules as the molecular effectors of cell-mediated lysis of target cells via apoptosis. Cytolytic T cell clones and TCR cDNA clones were also instrumental for the generation of TCR transgenic animals, which provided definitive evidence for negative selection of self-reactive immature thymocytes. In addition, studies of TCR complex signaling of immature thymocytes compared with mature T cells were consistent with the interpretation that negative selection occurs as a consequence of the incapacity of immature cells to produce IL-2, resulting in cytokine deprivation apoptosis. By comparison, taking advantage of cloned TCRs derived from T cell clones reactive with male-specific molecules, using TCR transgenic mice it was possible to document positive selection of female thymocytes when the male-specific molecules were absent. Focusing on the molecular mechanisms of T cell “help” for the generation of antibody-forming cells following the path opened by the elucidation of the IL-2 molecule, several groups were successful in the identification, isolation, and characterization of three new interleukin molecules (IL-4, IL-5, and IL-6) that promote the proliferation and differentiation of B cells. In addition, the identification of a B cell surface molecule (CD40) that augmented B cell antigen receptor-stimulated proliferation and differentiation led to the discovery of a T cell activation surface molecule that proved to be the CD40-ligand, thus finally providing a molecular explanation for “linked or cognate” recognition when T cells and B cells interact physically. Accordingly, the decade after the generation of the first T cell clones saw the elucidation of the molecular mechanisms of T cell cytotoxicity and T cell help, thereby expanding the number of molecules responsible for adaptive T cell immunity.

## TCR Signals

From the days of Nowell, T cell “activation” was synonymous with mitogen/antigen stimulation that resulted in T cell proliferation (1). Indeed, because tritiated thymidine (^3^H-TdR) incorporation was generally used as the read-out for T cell activation, it was assumed by everyone that the trigger for T cell proliferation resided solely with activation of the T cell antigen receptor (TCR) complex. However, with the realization that there is a molecular mechanism whereby TCR/CD3 signaling initiates proliferation, comprised of an endogenous hormone that triggers a hormone-like receptor, it became possible for the first time to begin to separate the biochemical signals generated by triggering the TCR from those triggered by the interleukin-2 receptor (IL-2R).

Deutsch first provided evidence that an early event in mitogenic triggering of lymphocytes might involve changes in the intracellular concentration and/or transport of cations (e.g., potassium, calcium) (2). Since the transmembrane electrical potential is an important factor in the movement of charged ions across the plasma membrane, Deutsch carefully measured it in human peripheral blood mononuclear cells (PBMCs), and found it to be between −35 and −52 mV. These results implied that ion gradients are maintained by energy-dependent processes in these cells, which were not generally considered excitable cells, like neurons.

Tsien subsequently took advantage of the newly available calcium-specific fluorescent indicator, Quin2, which made possible the first direct measurements of intracellular [Ca^2+^] in mouse thymocytes and pig lymph node cells using a spectrofluorometer (3). Mitogenic lectins such as phytohemagglutinin (PHA), Concanavalin-A (Con-A), and calcium ionophores (A23187, ionomycin) raised intracellular calcium concentration within a few minutes. Of note, deprivation of extracellular calcium prevented the response. Moreover, the lectin-induced intracellular [Ca^2+^] increases were sufficient to hyperpolarize the membrane potential, from −50 to −70 mV, which the authors speculated was likely due to a K^+^ conductance.

Nishizuka provided insight regarding the biochemical mechanism(s) whereby various cell surface receptors could transfer informational signals from the cell surface into the cellular interior with his path-breaking reports of two routes, protein kinase-C (PKC) activation and Ca^2+^ mobilization (4, 5). The signal-dependent breakdown of inositol phospholipids, particularly phosphatidylinositol bisphosphate (PIP_2_), was found to be the key event for initiating these processes. After stimulation of surface receptors, PIP_2_ is degraded immediately to produce 1,2-diacylglycerol and inositol 1,4,5-triphosphate (IP_3_). He showed that this turnover of membrane phospholipids is associated with an increase in intracellular [Ca^2+^], and the diacylglycerol produced initiates the activation of a calcium-dependent PKC. Thus, the extracellular receptor triggering information is transferred directly across the lipid membrane bilayer via breakdown of membrane phospholipids and ultimately results in intracellular protein phosphorylation.

Although these reports were suggestive that perhaps TCR/CD3 triggering could very well operate via this newly discovered transmembrane signal mechanism, the step from polyclonal mitogenic activation vs. monoclonal activation remained to be defined. Accordingly, Reinherz and collaborators used flow cytometry to analyze Quin2 fluorescence upon anti-TCR monoclonal antibody (MoAb) as well as anti-CD3 MoAb activation of T cell clones (6). When coupled to Sepharose beads, anti-TCR and anti-CD3 both caused a rapid increase in intracellular [Ca^2+^]. It is noteworthy that IL-2 did not cause any change in intracellular [Ca^2+^], despite promoting cellular proliferation. Also, removal of extracellular calcium using the chelator EGTA blocked anti-TCR and anti-CD3-induced proliferation of both cytolytic and helper/inducer T cell clones, but did not prevent IL-2-mediated proliferation.

Others took advantage of the human T cell leukemic cell line, JURKAT, which had been found to produce IL-2 when stimulated with mitogenic lectins (7, 8). Weiss working in John Stobo’s group showed that JURKAT clones would also produce IL-2 when activated by soluble anti-CD3, provided phorbol myristic acetate (PMA), which Nishizuka had shown to activate PKC, was also present (9). Weiss and coworkers then showed that soluble anti-CD3 MoAbs promoted a rapid increase in intracellular [Ca^2+^], as did PHA and calcium ionophore, and that the combination of PMA+ionophore promoted the production of IL-2 (10). Accordingly, these studies indicated that activation of both PKC and a rapid increase in intracellular [Ca^2+^] were required to promote IL-2 production, and taken together with Reinherz’s results, they indicated that solid phase but not soluble MoAbs reactive with either the antigen recognition chains of clone-specific TCRs or the pan-T cell signaling complex, CD3, activated both PKC and an increase in intracellular [Ca^2+^]. By comparison, IL-2 did not appear to operate via this pathway. Moreover, exactly what transpired between these early biochemical changes in T cells, which occurred within minutes of TCR/CD3 triggering, and the activation of IL-2 gene expression, which required at least an hour of continuous TCR/CD3 stimulation remained to be explored.

## Molecular Mechanisms of Cell-Mediated Cytolysis

Subsequent to the original description of cell-mediated lysis of ^51^Cr-labeled target cells (11), the mechanisms responsible for this phenomenon remained obscure, especially at the molecular level. However, Henkart’s team first showed via electron microscopy that human mononuclear cells punctured tubular holes into the membranes of susceptible target cells during antibody-dependent cellular cytotoxicity (ADCC), very similar to the morphology of complement-mediated lysis (12). Subsequently, Podack and Dennert found that cloned murine natural killer (NK) cells (13) and cloned alloantigen-specific murine IL-2-dependent CTL (14) produced similar circular membrane lesions in target cells that arose by membrane insertion of tubular complexes, which they conjectured were assembled from subunits during the cytolytic reaction, like the terminal components of complement. The tubules were detected on target cell membranes by immune electron microscopy and appeared to form transmembrane channels as seen in ultrathin sections.

To move toward a molecular explanation of these observations, Podack was ideally situated because he had already studied the complement components for several years with Muller-Eberhard. Even so, a major hurdle remained to be surmounted to move from these analytical morphological descriptions to a detailed molecular analysis of the phenomena. One of our perplexing findings noted as soon as we had successfully created the first cytotoxic T lymphocyte lines (CTLL) (15) and clones (16) was the appearance of the CTLL upon light and electron microscopy. The cytoplasm of the cells after methanol fixation and procession for staining by Wright–Giemsa revealed many granule-shaped cytoplasmic holes. Electron microscopy revealed that these organelles contained electron dense material. Podack hypothesized that these putative granules were the elusive cytotoxic granules of CTL, so that he took advantage of the capacity to grow up large numbers of CTLL-2 in IL-2-containing Con-A T cell supernatant (CATSUP). He generated >10^9^ cells that he used to isolate and purify the cytoplasmic granules. The plasma membranes were disrupted by N_2_ cavitation, and post nuclear supernatants were applied to self-generated Percoll density gradients, fractions of which were analyzed by structural analysis using electron microscopy, and for function by assaying for cytolytic activity in the presence and absence of calcium using sheep and rabbit erythrocytes as targets. The fractions containing very pure granules by EM yielded detectable cytolytic activity (17). SDS-PAGE of the fractions containing cytolytic activity revealed six characteristic bands, ranging in molecular sizes from 75 to 10 kDa under reducing conditions. This study represented the first demonstration of strong, calcium-dependent cytolytic activity of isolated CTL granules. Similar results for cytolytic granules isolated from large granular lymphocyte (LGL) tumor cells were reported by Pierre and Maryanna Henkart and their co-workers, so that it appeared that both CTL and NK cells lysed target cells using similar granules (18).

To approach the purification of the active proteins found in the cytolytic granules, Podack together with Young and Cohen (19), prepared 5 mL of packed cells from 6 L cultures of CTLL-2 cells containing 10–15% CATSUP. Granules with cytolytic activity purified from these packed cells via Percoll density-gradient centrifugation were mixed with 1 M phosphate buffer (pH 7.4), which precipitated the Percoll, allowing its easy removal by centrifugation. The transparent supernatant was then purified via gel filtration, fractions from which were assayed for protein and hemolytic activity, then pooled and further purified via ion exchange high pressure liquid chromatography (HPLC). Hemolytic fractions from this column were pooled and analyzed by SDS-PAGE, which yielded a single 75 kDa protein, which was provisionally designated protein-1 (P1). Analysis for hemolytic activity showed that lysis was Ca^2+^-dependent, inhibited by Zn^2+^ ions and accompanied by polymerization to form membrane-associated tubular complexes that formed large transmembrane pores, which caused rapid membrane depolarization in the presence of Ca^2+^. Masson and Tschopp, simultaneously purified a similar single protein from the cytolytic granules of another IL-2-dependent CTL line, terming this protein “perforin” (20).

These findings led to the inevitable comparison of P1/perforin to C9, the terminal lytic component of complement, and the conjecture that P1/perforin and C9 might have arisen from a common ancestral cytolytic protein that subsequently became specialized in cell-mediated and humoral cytolysis respectively. To pursue this hypothesis, Podack and his collaborators generated monospecific antisera reactive to both denatured and native C9 and P1/perforin (21). Cross-reactivity with P1/perforin and C9 was found using antisera reactive with denatured proteins but not by antisera reactive with native proteins, thereby indicating that the two proteins shared primary sequence epitopes, but not necessarily tertiary epitopes.

The next step, the identification and cloning of cDNA encoding P1/perforin, was accomplished by Podack’s team using the perforin-specific antisera and an expression λgt11 phage library constructed from murine CTLs (22). The predicted amino acid sequence contained 554 residues and as expected, displayed considerable homology with certain functional domains of C9, C8α, C8β, and C7. Also noteworthy, consistent with Reinherz’s finding of both CD8^+^ and CD4^+^ cytolytic T cell clones restricted to major histocompatibility complex (MHC) Class I and Class II encoded molecules, perforin mRNA was detectable in both class I and class II restricted cytolytic T cell clones, but not in non-cytolytic T cell clones or in other non-cytolytic cells. Thus, as predicted within a few years of the generation of monoclonal IL-2-dependent functional cytolytic T cells (16), an initial molecular mechanism whereby cytolytic T cells kill target cells became evident for the first time.

In parallel, other investigators pursued a somewhat different path to discern the molecular mechanisms responsible for CMC. Chang and Eisen reported that an irreversible protease inhibitor, *N*^α^-tosyl-l-lysyl-chloromethyl ketone (TLCK), markedly reduced MLC-generated CTL activity monitored by ^51^Cr release assays (23). Subsequently, Eisen’s group demonstrated that murine alloreactive IL-2-dependent CTL clones had remarkably high serine esterase activity in detergent lysates (24). By comparison, non-cytolytic cells contained little, if any esterase activity. Of interest, cytolytic activity from CTL lysates, but not intact CTL activity, was inhibited completely by the irreversible esterase inhibitors, DFP and PMSF, suggesting that normally the serine esterase was located internally and not expressed on the cell surface.

Independently a group led by Bleackley and another group led by Weissman used CTL clones to isolate and identify cDNA encoding novel T cell-specific serine proteases (25–27). Then, using the granules purified from two CTL clones, Masson and Tschopp isolated eight distinct serine proteases, designating them Granzymes A–H (28). Accordingly, the prophecy that normal CTL clones and their granules would allow the identification of the molecular mechanisms of lymphocyte-mediated cytolysis proved prescient (16). Even so, the identification of perforin/P1/cytolysin and Granzymes did not immediately answer the question as to how CTL and NK cells actually utilized these molecules to effect specific lysis of their target cells.

Apoptosis is a term from the Greek used to describe the “dropping off” or “falling off” of petals from flowers or leaves from trees. Kerr et al. coined the term to describe *“a general mechanism of controlled cell deletion, complimentary to mitosis in the regulation of cell populations”* (29). They described cellular apoptosis as structural changes initiated or inhibited by a variety of environmental stimuli, involving nuclear and cytoplasmic condensation and breaking up of the cell into a number of membrane-bound, ultrastructurally well-preserved fragments.

Russell and co-workers first showed that CMC led to release of both ^51^Cr from the cytoplasm and ^125^IUdR from the nucleus, whereas lysis of the same cells by antibody + complement or hypotonic shock led solely to the release of cytoplasmic ^51^Cr (30). Moreover, the intracellular disintegration of the nucleus occurred within minutes of CTL–target cell interaction, leading to two possible explanations, *“(1) The CTL injects degradative substances into the target cell, or (2) the target cell autolyzes itself in response to a signal from the CTL delivered at the plasma membrane”* (31).

Duke and co-workers summarized the data accumulated on the mechanisms responsible for CMC, which could be separated into three distinguishable stages (32). The first step involved target cell recognition and establishment of a stable effector:target cell contact or conjugate. This step required Mg^2+^ or Ca^2+^. The second step was found to be strictly Ca^2+^-dependent and constituted the “lethal hit” stage, during which the target cell was irreversibly committed to lysis. The third step involved effector cell independent target cell disintegration, wherein cytoplasmic macromolecules were released. Notably, all of these steps were found to be independent of protein and RNA synthesis, which were known attributes of apoptosis in other cell systems. Nevertheless, operating with the hypothesis that CMC might entail an apoptotic mechanism, these investigators showed for the first time that within minutes of exposure of target cells to antigen-specific CTL, their nuclear DNA began to fragment and preceded cytoplasmic ^51^Cr release by at least an hour. By comparison, killing mediated by heating, freeze/thawing, or lysing with antibody and complement did not yield DNA fragmentation. Furthermore, agarose gel electrophoresis of target cell DNA showed discrete multiples of ~200 bp subunits, a characteristic of apoptosis, “*suggesting that DNA fragmentation was the result of activation of a specific endonuclease”* (32). Despite these findings, these investigators pointed out that CMC-mediated DNA fragmentation differed from other examples of apoptosis, in that it did not require transcription or translation, presumably necessary to produce an endogenous nuclease.

One hypothesis that could serve to synthesize and reconcile all of these experimental data, including the inhibition of CMC by specific protease inhibitors, was that the CTL granules contained a pore-forming protein, perforin/cytolysin, which provided the conduit through which granule proteases, Granzymes, gained entrance to the target cell, liberating DNA, thereby leading to its rapid digestion. Accordingly, Henkart’s group provided the first data in support of this hypothesis: “*Although fully lytic concentrations of purified granule perforin/cytolysin alone failed to induce target cell DNA release, a combination of purified granzyme A and perforin/cytolysin induces substantial DNA release”* (33). Subsequently, Greenberg’s team reported similar findings from a serine esterase and cytolysin/Perforin purified from a rat NK tumor cell line (34). Consequently, two decades after the first description of ^51^Cr release (11), and a decade after the first description of cloned antigen-specific CTLs (16), the molecular mechanisms responsible for CMC became evident to everyone.

## Tolerance of Self via Negative Selection

In developing his theory of Clonal selection, Burnet necessarily had to account for the phenomenon of tolerance to self-bodily components that had been shown to develop during embryogenesis (35, 36).

Burnet stated:

“In the Burnet–Fenner theory (of antibody formation) (37), any potentialities of antibody production against body components were eliminated by the development of tolerance. In the present theory they are more readily disposed of by assuming that – at this particular stage of embryonic life – contact with the corresponding antigenic pattern results in the death of the cell. If the potential antigen persists long enough in high enough concentration, all clones which can produce this natural (auto) antibody will be eliminated” (38).

Of course, Burnet did not elaborate on the mechanism whereby self-reactive clones were eliminated, and this aspect of his theory had to be left to the future. Also, Burnet was writing before thymic-dependent maturation of T lymphocytes had been discovered (39–41), so that his view of immunity was restricted to humoral immunity. Even so, by definition, tolerance had to be antigen-specific, because it resulted in the capacity of the immune system to ignore self-antigens specifically. Accordingly, Burnet had to propose that antigen-specific self-reactive clones must be somehow deleted or otherwise inactivated, i.e., rendered anergic, during embryogenesis.

Further progress in this area had to await the identification of surface immunoglobulin as the molecular basis of the B cell antigen receptor (BCR) (42, 43), and the elucidation of the molecular nature of the TCR, which required another decade (44–46). Moreover, the generation of antibody-forming cells (AFCs) to most antigens was inextricably linked to “help” derived from T cells, so that the underlying molecular basis for the development of B cell tolerance necessarily depended on understanding T cell tolerance.

A breakthrough in the search for the mechanism(s) underlying T cell tolerance was reported by Kappler and colleagues, who generated a MoAb that reacted with all T cells that expressed TCRs encoded by β-chain variable gene segments specified by the variable gene region Vβ17a (47). Moreover, T cells bearing Vβ17a-encoded receptors that reacted with cells that expressed the MHC Class II protein, IE. Using this MoAb, it was found that T cells expressing Vβ17a were selectively eliminated from the mature thymocyte and peripheral T cell populations of mice expressing MHC class II IE, but were present in the expected numbers in immature thymocyte populations (48). These observations were consistent with the notion, as proposed originally by Burnet, that tolerance to self-antigens (in this case self-MHC-encoded molecules) is due to clonal elimination. In addition, their findings indicated that the elimination of self-reactive thymocytes occurred in the thymus as immature thymocytes were selected to enter the mature thymocyte pool. All of these experiments were possible because the Vβ17a TCRs amounted to as much as 10–20% of all the TCRs.

Despite these provocative findings, it still remained unclear whether this phenomenon was restricted to recognition of MHC-encoded self-molecules, or indeed whether other so-called “nominal” self-antigens associated with MHC-encoded molecules would also react with particular TCR Vβ gene segments. Subsequently, in the course of screening peripheral T cells and mature cortisone-resistant thymocytes from various inbred mouse strains using a MoAb that recognized all TCRs encoded by Vβ6 gene segments, MacDonald and coworkers made the serendipitous observation that Vβ6+ T cells correlated negatively with the expression of one allele of the “minor lymphocyte-stimulating” *(Mls)* gene locus in all inbred mouse strains tested (49). At the time of these studies, the structure and function of the *Mls* gene product(s) were unknown. However, *Mls* was remarkable, in that it was the only antigen other than the MHC-encoded molecules that could specifically stimulate proliferation of a high proportion of lymphocytes from unimmunized mice. These findings were interpreted as providing *“unequivocal evidence that expression of a particular V*β *segment confers preferential T cell reactivity in vitro to Mls-encoded determinants and that TCRs using V*β*6 are selected against in the thymus of mice that express Mls-encoded gene products.”* Thus, they felt that, consistent with Burnet’s proposal, tolerance is mediated by the deletion of self-reactive clones rather than by alternative mechanisms such as anergy or peripheral suppression. Very similar findings were reported simultaneously by Kappler and coworkers (50).

Although these reports were certainly consistent with Burnet’s conjecture that tolerance results from the deletion of immature autoreactive cells, because specific peptide antigen-reactive T cells had not been shown to be deleted, the question remained open. Obviously, the problem was the detection of very scarce antigen-reactive cells, made impossible by the tremendous diversity of antigen recognition. The development of transgenic mice first by Ruddle’s group (51), and then by Brinster and coworkers (52, 53) provided the necessary tools to analyze the mechanisms responsible for self-tolerance to specific peptide epitopes *in vivo*. Thus, von Boehmer and team took advantage of monoclonal T cells cytolytic for peptides specified by the male minor histocompatibility antigen (HY) that they had generated using female T cells stimulated by male T cells *in vitro*, and cloned by limiting dilution using IL-2-containing media (54) (see Toward a Molecular Understanding of Adaptive Immunity: A Chronology, Part II). They chose one of their clones for study because it’s TCRβ-chain could be recognized by a MoAb specific for all three members of the Vβ8 gene family (55). They then injected fertilized eggs with genomic DNA harboring the productively rearranged TCR α and β genes isolated from the HY-specific cytolytic T cell clone (56). The transgenic founder mouse contained four copies of the TCR-α and two copies of the TCR-β transgenes integrated on the same chromosome. Noteworthy, the presence of the transgenes inhibited the expression of the endogenous α and β TCR genes, with the result that the majority of T cells expressed the transgenes (55). Thus, they essentially created *in vivo* clonal T cells, thereby circumventing the complex TCR repertoire, so that the fate of transgenic T cells expressing only one TCR complex reactive with a single peptide could be studied.

T cells with the phenotype of the cytolytic HY-specific clone were found to be frequent in female, but not in male transgenic offspring, despite the fact that peripheral T cells in animals of both sexes expressed both transgenes. Peripheral T cells in male, but not female mice, had an abnormal CD4/CD8 phenotype, in that over 90% of T cells in the lymph nodes of male transgenic mice lacked the expression of both CD4 and CD8, or expressed very low levels of CD8. Moreover, there were very few CD4^+^CD8^−^ T cells. This unusual phenotype of peripheral male T cells was a consequence of the deletion of autospecific HY-reactive thymocytes that expressed high densities of CD8 molecules, predominantly cortical CD4^+^CD8^+^ immature thymocytes. Therefore, these data supported the interpretation that double-positive cortical thymocytes are precursors of single-positive mature thymocytes, and that the accessory molecules CD4 and CD8 are important for the deletional process. Moreover, these data first established that negative selection of self peptide-specific T cells occurs as a consequence of TCR recognition of self-peptide.

Accordingly, all of these data confirmed Burnet’s prediction that tolerance to self-autoantigens, at least in the T cell compartment, occurs via the deletion of cells capable of recognizing self-peptides. Moreover, the data from the TCR transgenic mouse experiments were especially convincing that the TCR *and* the accessory molecules, CD4 and CD8, which confer antigenic specificity and sensitivity, were responsible for initiating the deletional process. However, these experiments did not yield any insight into the molecular mechanism(s) operative. In other words, these data revealed “what happens,” but they did not provide any clues as to “how it happens.”

## Negative Selection: Roles of the TCR vs. IL-2 in Triggering Apoptosis

Almost simultaneously, as experiments were reported by investigators focused on genetic approaches in tolerance induction, others were exploring biochemical and immunological approaches to understand T cell maturation and selection in the thymus. Experiments reported by Raulet using flow cytometric analysis revealed that a small thymocyte subpopulation lacks expression of both CD4 and CD8 TCR accessory molecules, and contains the most immature cells (~3% of adult thymocytes). As MoAbs capable of recognizing what is now known as the IL-2Rα chain became available (57–59), ~1/3 of this “double-negative” thymocyte subpopulation, or only 1% of all thymocytes, were found to express this cytokine receptor chain (60). In addition, double-negative fetal thymocytes were also positive for IL-2Rα chain expression. Even so, it is noteworthy that when exposed to purified recombinant human IL-2 (rhIL-2), the immature double-negative thymocytes proliferated poorly, thus questioning whether most of these immature IL-2Rα+ thymocytes expressed a functional IL-2R, and also whether IL-2 itself was involved in thymocyte proliferation and differentiation. This was especially cogent, given that Con-A without IL-2 supplementation was ineffective in stimulating thymocyte proliferation, indicating that immature thymocytes could not produce IL-2. Moreover, IL-2Rs were undetectable on the double-positive thymocytes subset, which makes up the majority of immature thymocytes, ~90% and the stage where negative selection appeared to be taking place.

From the earliest experiments with IL-2-dependent CTLL, it was observed that IL-2 deprivation for even a few hours would lead to irreversible cell death, although the mechanism(s) responsible were totally obscure (61). Accordingly, of interest soon after Raulet’s thymocyte experiments, Duke and Cohen reported that IL-2 withdrawal from IL-2-dependent proliferating CTLL and helper T cells results in extensive chromatin cleavage that precedes plasma membrane breakdown by several hours (62). The DNA fragments observed were not randomly generated but consisted of regular sized oligonucleosomes. Noteworthy was the finding that DNA fragmentation, like cell death, did not occur in the presence of protein synthesis inhibitors, suggesting that IL-2 deprivation leads to the activation of the expression of an endogenous endonuclease specific for linker DNA. All of these data indicated that IL-2-deprivation cytolysis occurs via a suicide program with all of the classic signs of apoptosis.

A subsequent analysis of murine thymocyte expression of CD3, CD4, and CD8, and reactivity to stimulation via anti-CD3 by Allison and coworkers revealed that 80% of adult thymocytes express both CD4 and CD8 (i.e., double-positive), 3% express neither (double-negative), 12% only express CD4 (CD4 single-positive, and 3% expressed CD8 alone) (CD8 single-positive) (63). Noteworthy, there was a distinct difference in CD3 density among the subsets, with the single-positive cells expressing the highest density, while the double-positive cells expressed only a low density of CD3. In this report, about half of the double-positive cells expressed detectable CD3. To assess the functionality of the CD3 signaling molecules expressed by double-positive thymocytes, by comparison with single-positive peripheral T cells isolated from the spleen, single cell intracellular calcium concentrations were measured using flow cytometry. It was obvious that the anti-CD3-triggered increased intracellular Ca^2+^ concentrations of double-positive thymocytes were only about half of the mature peripheral splenic T cells. However, PKC, like calcium second messengers a product of CD3-induced phosphatidylinositol 4,5-bisphosphate hydrolysis, was activated by anti-CD3 stimulation of murine double-positive thymocytes.

As the components of the early steps of T cell activation appeared to be functional in double-positive thymocytes, the expression of the IL-2Rα-chain and the production of IL-2 were assessed next (63). In contrast to mature peripheral single-positive splenic T cells, the double-positive thymocytes failed to produce IL-2 or to express the IL-2Rα-chain, and to proliferate, even if irradiated macrophages to serve as a source of IL-1 were present. Also, noteworthy was the lack of a proliferative response of the double-positive thymocytes, even if IL-2 was added exogenously, thereby indicating the lack of functional IL-2R expression in response to anti-CD3 stimulation. Accordingly, the CD4CD8 double-positive thymocytes undergo negative selection when triggered via their TCR/CD3 complex by self-peptide/MHC complexes *in vivo*, but the TCR/CD3 complex expressed by these cells appears immature, incapable of promoting transcription and translation of genes such as IL-2 and the IL-2Rα-chain, which are required for a proliferative response by mature peripheral single-positive T cells *in vitro*. Thus, a plausible mechanism for the apoptosis underlying negative selection is the lack of IL-2 production and IL-2R expression by TCR/CD3-triggered immature double-positive thymocytes, in that without the IL-2-derived pro-survival and proliferative/differentiative signals, premature TCR/CD3 triggering may well activate apoptosis as a default pathway.

These data were confirmed and underscored by Reinherz’s group who studied human thymocytes compared with peripheral mature T cells (64). Using the unique observation that CD3^−^ thymocytes could be triggered to proliferate via activation by MoAbs reactive with anti-CD2, they found that anti-CD3 would actually inhibit this proliferative response. In examining this phenomenon further, they found that CD3^+^ thymocytes, like their murine counterparts, could not be triggered to proliferate, and that anti-CD3 stimulation did not promote IL-2 production, but did lead to IL-2R expression. Moreover, anti-CD3 activation actually suppressed IL-2 gene expression stimulated by anti-CD2. Thus, by comparison with mature peripheral T cells, triggering CD3^+^ thymocytes with anti-CD3 leads to a rapid *suppression* of IL-2 gene expression, thereby preventing the cells from receiving the pro-survival and proliferative effects of IL-2, thus creating a situation imitating cytokine withdrawal apoptosis. Therefore, TCR/CD3 activation of immature thymocytes results in an active suppression of IL-2 gene expression, not simply a passive lack of IL-2 gene activation. This begs the question as to the mechanism responsible, which remained obscure.

This conjecture was supported by Jenkinson and colleagues, who studied the effects of anti-CD3 on thymocytes in fetal organ culture, which provides an *in vitro* system that can support TCR gene rearrangement and expression, and avoids the problem of low viability encountered in cultures of isolated thymocytes (65). They found that anti-CD3 added for the last 18 h of culture resulted in a substantial reduction in cell yield (~43%), and the chromatin condensation and cell shrinkage characteristic of apoptotic cells detectable by electron microscopy, as well as the degradation of DNA to oligonucleosomes characteristic of apoptosis. Similar results were obtained using a MoAb reactive with TCR Vβ8, as well as a super-antigen, staphylococcal enterotoxin-B, reactive with TCR Vβ8+ cells. Accordingly, these data are all consistent with the conclusion that triggering of immature CD4CD8 double-positive thymocytes either via MoAbs reactive with CD3, or MoAbs and superantigens reactive with specific TCR Vβ8+ cells, results in apoptosis rather than activation (66).

## Positive Selection-MHC and Endogenous Peptide

A picture is worth a thousand words, they say, and thus the determination of the structure of human Class I histocompatibility leukocyte antigen (HLA) from crystallized protein by Bjorkman, a student working with Wiley and Strominger, rocked the immunological community (67). Since the pioneering experiments of Schlossman (68) and path-breaking findings of Unanue and Askonas more than 20 years earlier (69), it had become established that T cells recognize short peptides (~6–20 residues), digested and presented by antigen presenting cells, somehow complexed to molecules encoded by genes of the MHC.

However, without any knowledge of the structure of these molecules, it was difficult to imagine how the myriad of foreign peptides (estimates of ~10^7)^ could interact with each MHC encoded molecule, and furthermore how a single TCR recognized or interacted with the peptide–MHC complex. Accordingly, the shock from visualizing the first HLA molecular structure was finding the peptide-binding groove situated between two alpha helices (67). Also, finding the groove to be already occupied with what ultimately turned out to be an endogenous peptide that persisted throughout the entire purification and crystallization procedure suggested that a peptide, either self or non-self, is always bound to the histocompatibility molecules (70).

Thus, by 1987 it was established that a single TCR recognizes a single peptide–MHC complex, and as a consequence of the work of Bjorkman and colleagues, it was finally understood exactly how peptides complexed with the MHC-encoded molecules to form a single peptide/MHC antigen recognition molecule. However, even before the discovery of MHC restriction of T cell antigen recognition, Jerne had proposed that the germ cells of an animal carry a set of genes encoding the combining sites of lymphocyte receptors which he hypothesized were directed against a complete set of MHC-encoded antigens of the species to which the animal belongs (71). Subsequently, it was reported that precursor T cells differentiate recognition structures for self-MHC molecules expressed on the thymic epithelial cells (72, 73). However, exactly how this phenomenon occurred remained obscure.

The two subsets of T cells distinguished by their expression of either CD4 or CD8 molecules were first delineated by Reinherz’s group, who studied human PBMCs (74, 75), and then human IL-2-dependent cytolytic CD8^+^ and CD4^+^ T cell clones (76). Several years after the work with human T cells, a MoAb that reacts with the murine equivalent of CD4 was developed by Fitch’s group, allowing Swain to confirm the correlation between CD4 expression and MHC class II restriction of antigen recognition in the murine system (77). Analysis of all of Reinherz’s human T cell clones revealed CD8^+^ T cells to be MHC Class I restricted, whereas all CD4^+^ T cell clones were MHC Class II restricted in their capacity to recognize antigens (76). Noteworthy from these data was the observation that both MHC Class I restricted and MHC Class II restricted T cell clones were capable of cytolysis as determined by the standard 4-h ^51^Cr release assay. However, in addition, CD4^+^ MHC Class II restricted T cell clones could also provide T cell “help” for antibody production, and thus could be “helper” T cells (Th). Accordingly, although cytolytic T cells (Tc) were traditionally considered CD8^+^ while Th cells considered CD4^+^, this discrete separation was not supported by the data derived from T cell clones (76). Instead, CD8 expression correlated with MHC Class I antigen recognition, while CD4 expression correlated with MHC Class II antigen recognition.

Thus, it was proposed that mature peripheral thymic-derived cells are somehow “positively selected” from immature thymocytes by MHC-encoded molecules expressed on thymic stromal cells, and upon emerging from the thymus, the TCRs of these cells would bind foreign peptide antigens that are already bound to self-MHC encoded molecules. Accordingly, should this be the case, von Boehmer hypothesized that in the absence of foreign peptides during thymopoiesis, the interaction of the TCR with Class II and Class I thymic self-MHC molecules should direct or select for the differentiation of immature T cells into single-positive CD4^+^ and CD8^+^ mature T cells respectively (78). Thus, von Boehmer’s team focused on female TCR transgenic mice that recognized the male-specific HY antigen, because this antigen was absent during thymopoiesis in these animals. Theoretically, female mice should “positively select” CD8^+^ T cells capable of recognizing HY peptide/self-MHC, but not self-MHC without HY peptide, or HY peptide and a third party Class I MHC. Their results were consistent with this hypothesis, i.e., that the interaction of Class I MHC-encoded molecules in the thymus with the αβ heterodimeric TCR determines the CD4/CD8 phenotype of mature T cells in the absence of reactive foreign peptide (78).

To investigate further the specificity of the “positive selection” exerted by MHC-encoded molecules, the von Boehmer team performed thymus repopulation experiments (79): lethally irradiated females of recombinant strains that differed at D and K through to the S sub-regions of the MHC, were transplanted with bone marrow cells from H-2^b^ TCR αβ HY-specific transgenic female mice. Five weeks after transplantation, the repopulating capacity of the donor stem cells, as estimated from the cellularity of the thymuses of the recipients, correlated with the degree of compatibility within the MHC: the highest numbers of thymocytes (~5 × 10^7^) were recovered from syngeneic and semi-allogeneic recipients, the lowest (~8 × 10^6^) from allogeneic recipients differing across the whole MHC. These investigators went on to hypothesize *“that the specific low affinity interaction of the TCR with thymic stromal MHC-encoded molecules is sufficient to select immature T cells but insufficient to activate mature T cells. The specific low affinity interaction in the thymus may be complemented by accessory molecules* (CD4/CD8 co-receptors) *present on either immature thymocytes or selecting thymic stromal cells, or both. Such a complementation could then lead to the positive selection of T cells with low affinity for self-MHC*.”

Entirely consistent with these findings and hypothesis, von Boehmer’s group followed up their *in vivo* findings with *in vitro* experiments using double-positive thymocytes from female H-2^b^ TCR αβ HY-specific transgenic mice. When exposed to thymic or splenic adherent cells from male H-2^b^ mice during 24–48 h of suspension culture, deletion of double-positive female thymocytes occurred only in the presence of cells bearing HY antigen with the appropriate (H-2^b^) MHC-encoded molecules. These findings indicated that the deletion of double-positive thymocytes is not a unique property of the thymic microenvironment, but is the property of a certain developmental stage of T cells, which makes it sensitive to deletion. Thus, female double-positive thymocytes will be deleted when exposed to the HY-antigen presented by the proper MHC-encoded molecules *in vitro*, just as the male double-positive thymocytes are deleted *in vivo*, when exposed to their own self-antigen. Also noteworthy, is the fact that female single-positive CD8^+^ mature peripheral T cells do not undergo apoptosis when confronted with HY+ APCs. Rather, they undergo activation and clonal expansion, underscoring the interpretation that immature double-positive thymocytes are negatively selected because of their immaturity.

It is also noteworthy that the hypothesis to explain these findings of positive and negative selection, i.e., that there must be a difference in the *affinity* of self-antigen stimulation of the TCR-co-accessory molecular complex to account for such disparate fates of life vs. death, remained untested at the time in the early 90s. A further, unexplored aspect of positive selection is the question of whether, like negative selection, a self-peptide occupies the groove of the MHC-encoded molecules in the thymus, such that all positively selected T cells that exit the thymus and populate the periphery have the capacity of reacting to self, should the self-peptide concentration become high enough to surpass a putative threshold, leading to T cell activation, proliferative clonal expansion and differentiation to become effector cells capable of autoimmune reactivity. Alternatively, perhaps there is a peripheral tolerance mechanism that functions to prevent auto-recognition and activation. These considerations would necessarily be the subjects of future investigations.

## Molecular Mechanisms of T Cell “Help”

One of the purposes of the creation of the interleukin nomenclature, whereby the molecules were simply numbered, was the anticipation of additional new and novel interleukins yet to be discovered. In this regard, murine Th clones had already been generated using CATSUP by Schreier and co-workers (80, 81). Thus, operating with the hypothesis that there must be a Th cell-specific growth factor or interleukin similar but different from the CTL-specific IL-2, Hapel working in Ihle’s group, reported that a new and novel cytokine promoted the proliferation and differentiation of immature hematopoietic precursor cells of athymic (*nu/nu*) mice to become Lyt-1+Lyt-2− Th cells (82). However, these investigators appeared to be unaware that the Lyt-1 alloantigen, which had been thought to mark Th cells, had been found on all T cells, so that it did not specify the Th subset as did the CD4 molecule expressed by human T cells (83, 84). In any event, since IL-2 appeared to preferentially lead to the growth of CTL at the time, it seemed reasonable that there might also by a Th-specific growth and differentiation factor in CATSUP. Ihle’s group had reported that conditioned media from mitogen- or alloantigen-stimulated lymphocytes promoted the expression of 20α-hydroxysteroid dehydrogenase, which had been reported to be associated with mature but not immature T cells (85, 86). Because this activity appeared to target immature hematopoietic cells, and because the initial biochemical characteristics of the activity appeared to be different from those reported for both IL-1 and IL-2, Ihle’s group proposed that the activity be termed IL-3. Of course, all of this occurred before any cytokine activity had been purified to homogeneity and attributed to a single molecule (see “Toward a Molecular Understanding of Adaptive Immunity: A Chronology, Part I and II”), so this nomenclature proposal was problematic.

Unbeknownst to Hapel and Ihle, Schrader had also reported that CATSUP promoted the outgrowth of immunoglobulin-negative, Thy-1-negative, and Lyt-1/Lyt-2-negative non-adherant cells from murine splenocytes, which he termed “persisting cells” (P-cells) (87). By comparison with the lack of these T cell markers, MHC-encoded Class I and II molecules as well as Fc receptors were readily detected on these cells. In other experiments, metachromatic granules were found in these cells, and they were found to contain histamine, suggesting that the P-cells were from the mast cell lineage (88). Schrader went on to show that the activity that he found in CATSUP was similar to the colony stimulating activity (CSA) described by Metcalf and colleagues that promoted the growth of mixed hematopoietic colonies of erythroid and myeloid cells, but was distinct from T cell growth factor (TCGF) and T cell-derived granulocyte-macrophage colony-stimulating factor (GM-CSF) (89).

Soon thereafter, Hapel and coworkers reported the cloning of a cDNA encoding murine IL-3, which they identified by the growth promoting effects of oocyte-translated mRNA on an IL-3-dependent cell line 32D clone-23 created by Ihle’s group (90). The nucleotide sequence predicted an amino acid sequence with a molecular size of 15,102 Da, so that it was similar but different from IL-2 (91, 92). Hapel’s group then collaborated with Metcalf’s group to show that the molecularly cloned IL-3 induced 2-α-hydroxysteroid dehydrogenase activity in splenic lymphocytes from athymic (*nu/nu)* mice, in addition to proliferative activity for 32D clone-23 and FDC-P1 cell lines, and CSA for granulocyte-macrophage, eosinophil, megakaryocyte, NK-like, erythroid, and multipotential colony-forming cells from murine fetal liver and adult bone marrow (93). Thus, IL-3 was shown to promote multipotential hematopoietic stem cell proliferation, and not to be a Th-specific growth factor.

As detailed in Part I of “Toward a Molecular Understanding of Adaptive Immunity: A Chronology” (94), at least three cell types were found to be required for the generation of AFCs *in vitro*; B cells, T cells, and macrophages. Mitchison had shown that there appeared to be a “linked or cognate recognition” between B cells and helper T cells important for the mechanism of T cell help leading to the generation of AFCs (95). Also, the contribution of both macrophages and T cells could largely be replaced by supernatants from these cultured cells (96, 97), so that there seemed to be both cell-associated and soluble components to the “help” that B cells received from T cells and macrophages. Subsequently, during the 70s many purported to show either macrophage- or T cell-derived soluble helper activities that promoted the generation of AFCs, either by promoting B cell proliferation or Ig secretion or both. However, because of the complexities of target cell populations used in the bioassays, as well as the difficulties of protein analysis and purification, and the intricacies of monitoring both proliferation and Ig secretion, the field did not progress for more than a decade.

With the advent of the generation of both cytolytic and helper T cell clones (16, 81), the stage was set for new approaches of reductionist science to approach the question of the molecular regulation of AFC generation and the contributions of both macrophages and T cells. In particular, Schreier and coworkers generated multiple Th clones specific for sheep red blood cells (SRC) or horse red blood cells (HRC) as antigens that were amenable to the analysis of their effects on AFCs using the Jerne plaque assay (98) and the Mishell–Dutton system (99). Early on, they established that antigen-specific Th cell clones, propagated in culture in serum-free medium for up to 15 months, would enable *nu/nu* mice to respond to T-dependent antigens *in vivo* by producing specific antibodies and the formation of as much as five times the number of AFCs as expected in a primary response of normal mice (81).

Schreier then went on to examine the nature of T cell help. In a detailed analysis of T cell help using Th clones, and as targets either LPS-activated B cell blasts or isolated small resting B cells, they found that Th clones released an antigen/APC-activated MHC Class II restricted “soluble helper factor” that promoted LPS-induced B cell blast proliferation and Ig secretion (100, 101). This soluble “T cell helper factor” was effective regardless of the MHC or antigen specificity of the B cell blasts. By comparison, small resting B cells required the physical presence of Th clones, as well as their antigen/MHC-restricted antigen-non-specific secreted soluble Th factor to undergo proliferative expansion and Ig secretion. It appeared that small resting B cells needed two signals, one derived from the Th-B cell contact from Th antigen “linked recognition” to become responsive to the soluble Th factor, which then promoted proliferation.

Several aspects of these results warrant analysis. First, the generation of B cell blasts for use as target cells introduced an unknown, in that at the time the nature of the LPS-induced blastogenesis and proliferation remained obscure. It was not at all clear why LPS was a B cell mitogen in the mouse but not in the human (102), and whether LPS mimicked antigen-activation or not. Second, these experiments were performed before the molecular nature of the TCR was realized, and before the nature of peptide antigen binding to MHC-encoded molecules on APCs was known. Thus these investigators conjectured that the “linked or cognate” antigen recognition occurred via antigen bound to surface Ig on the B cell (BCR) and antigen bound to the TCR on the surface of the T cell, as proposed originally (95). However in addition, now they suggested that, a second signal was supplied to the B cell by the T cell, via the elusive second TCR interacting with B cell Class II MHC-encoded molecules. Despite these complexities and unknowns, the data generated using Th clones and B cell blasts vs. small resting B cells were irrefutable and had to be explained.

The cellular source, as well as the molecular nature of Schreier’s antigen-non-specific B cell replication and maturation factor (BRMF) was obscure. Thus, the investigators stated that the cellular source of the factor *“may be either the antigen-specific helper T cell or the macrophages that interact with the helper T cell.”* Also, “*We imply that one factor* (molecule) *induces both maturation* (i.e., Ig production) *and* (DNA) *replication, although it is equally possible that separate factors* (molecules) *govern these two reactions of a B cell.”* This same interpretation was applied in a separate report that explored the various soluble factors produced by Th clones (103). When Th clones were stimulated by specific antigen and histocompatible APCs, several apparently distinct activities appeared in culture supernatants that promoted colony formation of various hematopoietic cells, including macrophages, granulocytes, erythroid cells, as well as TCGF and BCGF activities. However, even though Th clones were used rather than heterogeneous T cell populations, because heterogeneous APCs + antigens were required to elicit the activities, the cellular source of each of these activities remained unknown, i.e., whether T cell or macrophage-derived, and also whether there were many or just one cell and molecule responsible.

Taking a page from the TCGF/IL-2 experience, the use of cloned Th cells at least solved the problem of the identity of the T cells used to produce the conditioned media containing the active molecules. However, long-term normal B cell lines and clones had yet to be developed, so that the target B cells used for the bioassays were still heterogeneous. Therefore, the next best approach was to use B cell blasts obtained using activation with B cell mitogens, or alternatively, B lymphoma cells, which could be cloned. Pure working in Vitetta’s group, in collaboration with Dennert, Swain, Dutton, and Takatsu, studied the effects of T cell supernatants obtained from a normal alloantigen-specific T cell line, as well as Con-A-activated T cell lymphoma and hybridoma cells, on the proliferation and polyclonal IgM production by anti-IgM/D-activated neonatal and adult splenocytes as well as BCL_1_ lymphoma cells (104). They found that Sepharose-bound anti-Ig induced the proliferation of adult but not neonatal B cells, and that IgM production required T cell supernatants, which induced the production of IgM by both normal (neonatal and adult) and neoplastic B cells. These data seemed to indicate that BCR stimulation led to proliferation, but that differentiation to Ig production depended upon factors in the T cell supernatants.

In a separate report, Swain and Dutton examined T cell replacing factor (TRF) activity derived from the same alloantigen-stimulated long-term T cell line generated by Dennert (105). Using the classic TRF assay of Schimpl and Wecker, comprised of SRBCs cultured with T- and macrophage-depleted splenocytes (97) as a source of target B cells, they found that supernatants from this T cell line had TRF activity but not IL-1 or IL-2 activity. Of note, this TRF supernatant synergized with IL-2 in the generation of AFCs, which was especially evident when the two activities were titrated. They speculated that this activity could be the “late-acting TRF,” originally described by Schimpl and Wecker, which was postulated to replace the requirement for T cells by promoting the differentiation of antigen-stimulated B cells, facilitating the production of IgM. However, these investigators rightly discussed the problems associated with the interpretations of their experiments, which used heterogeneous cell populations and T cell lines instead of cloned cells. For example, any soluble activity might amplify residual T cells in the purified B cell population instead of working directly on the B cells themselves. In addition, at this time no biochemical characterizations of the TRF activity were reported.

Soon thereafter, Krammer and Vitetta reported evidence for “B cell differentiation factor(s)” (BCDFs), which appeared to promote B cells to switch isoptypes from IgM to IgG (from ~1 to ~20% of AFCs), and especially to IgG_1_ (from ~40 to ~90%) (106). As targets, they used LPS splenic B cell blasts purified by negative selection with monoclonal anti-Thy1 + complement (C’), and supernatants from two long-term IL-2-dependent alloreactive T cell lines stimulated with Con-A. B cells cultured for 6 days with LPS and T cell supernatant wielded IgG AFCs, whereas no IgG AFCs were produced by LPS alone, only IgM AFCs. These T cell supernatants promoted the appearance of IgG from fluorescent activated cell sorter (FACS)-selected IgG-negative B cells, and furthermore, a switch from IgG_2a_ and IgG_2b_/IgG_3_ to predominantly IgG_1_ (~90%) occurred. They speculated that the LPS-dependence of Ig production of any isotype at all suggested the necessity for a specific signal that could have been a proliferative signal or one that induced the expression of receptors for the BCDF(s). By means of the traditional IL-2 and TRF assays it was determined that the supernatants from the two T cell lines were negative, so that it was concluded that they had identified a new activity, even though they provided no data regarding molecular characteristics.

Simultaneously, Howard working in Paul’s group, in collaboration with Farrar and Takatsu, reported the identification of a T cell-derived B cell growth factor (BCGF) distinct from IL-2 (107), based on a system originally reported by Parker (108). By comparison with the assays of others, this group used as targets negatively selected (various antibodies reactive with T cells + C) splenic B cells activated for 72 h with affinity-purified goat anti-mouse IgM. For a source of T cell activities, supernatants were obtained from a mouse thymoma (EL4) and a T cell hybridoma activated with PMA for 48 h. PMA was removed from the supernatants by absorption with activated charcoal. As in the IL-2 bioassay, the relative concentrations of BCGF activities were compared by the reciprocal of the dilution that yielded 50% of maximal activity. When 50 μg/mL of anti-IgM was used with 5 × 10^5^ cells/microwell, an easily detectable proliferative response could be quantified with ^3^H-TdR incorporation without the addition of a T cell supernatant. However, at 10-fold lower anti-IgM concentrations and cell densities, T cell supernatants were required to promote detectable ^3^H-TdR incorporation. To exclude the possibility that IL-2 was responsible for the BCGF activity, the supernatants were absorbed with cloned IL-2-dependent T cells. Moreover, molecular gel filtration of the supernatants yielded distinct peaks of IL-2 and BCGF activities. Both partially purified IL-2 and BCGF failed to replace the requirement for T cells (TRF activity) in the generation of AFCs from purified B cells + SRBCs. However, the inclusion of all three activities (IL-2, BCGF, and TRF) did yield AFCs. Thus, these investigators concluded that there were three activities (TCGF, BCGF, and TRF) that functioned independently to promote B cell proliferation and differentiation to AFCs. Like the model of T cell activation and proliferation (109, 110), these investigators proposed *“that anti-IgM delivers a non-proliferative signal to resting B cells, possibly causing the expression of receptors for BCGF; and that BCGF delivers a proliferative signal to these anti-Ig activated growth factor-sensitive B cells.”*

In collaboration with Mizel and Lachman, Howard and Paul went on to show that in addition to triggering the BCRs, optimal B cell proliferation required both a T cell-derived BCGF and a late-acting signal derived from purified IL-1 (111). From their experiments, it was shown that this combination would only result in B cell proliferation and not Ig secretion, and furthermore that the target B cells had to be cultured at low cell densities (5 × 10^4^ cells/well or 2.5 × 10^5^ cells/mL) for the BCGF and IL-1 effects to become detectable. It did not appear that the IL-1 acted by stimulating residual T cells to produce another BCGF, similar to the effect of IL-1 on T cells to produce IL-2 (112, 113), but the mechanism of the IL-1 B cell proliferative effect remained unexplored.

Swain and Dutton then collaborated with Howard, as well as Kappler, Marrack, and Watson, to try to discern whether there were several or only one T cell-derived BCGF (114). They found that supernatants derived from the EL4 thymoma synergized with anti-Ig in promoting the proliferation of normal splenic B cells, but failed to have activity when the BCL_1_ lymphoma cells were the target. This activity they termed BCGF-I. By comparison, supernatants from the Dennert alloreactive T cell line had reciprocal activities, promoting the proliferation of BCL_1_ cells, but not anti-Ig-stimulated normal splenic B cells (BCGF-II). They discussed the conundrum of wondering whether there were different states of B cell differentiation responsive to different growth factors, or whether there were different subsets of B cells involved. Again, a molecular characterization of the activities was lacking. However, they went on to show that homogeneous, MoAb-purified IL-2 acted on an antigen-specific (ovalbumin) T cell line to produce BCGF-I, thereby indicating that the various cytokine activities found in antigen-activated T cell supernatants could very well be interdependent (115).

Sideras working in Severinson’s group on the IgG_1_-inducing factor, constructed a quantitative bioassay patterned after the TCGF bioassay using normal LPS-stimulated splenocytes as target cells and isotype-specific AFCs as the indicators (116). This assay yielded parallel dose-response curves identical to those described for the TCGF assay, and the relative concentrations obtained from different IL-2-dependent cloned alloreactive T cells or hybridomas stimulated with Con-A could be ascertained by the 50% AFC responses. Using this assay, they found that the IgG_1_-inducing activity, and the IgG_3_/IgG_2b_ reducing activities, co-migrated both quantitatively and qualitatively, thereby suggesting that they were attributable to the same lymphokine activity. Furthermore, preparations of BCGF-I, purified via MoAb affinity (117) scored positive in their IgG_1_-inducing bioassay, as well as the IgG_1_-inducing assay of Vitetta. Accordingly, these data suggested that a single molecule might be responsible for both B cell proliferative (BCGF) and differentiative (BCDF) activities. Moreover, the biochemical properties of this activity differed from those reported for IL-1, IL-2, IL-3, CSA, IFN_γ_, BCGF-II, and TRF (118).

To proceed beyond these experimental approaches, Severinson collaborated with Honjo’s group to identify a cDNA encoding a protein with IgG_1_-inducing activity (119). The cDNA predicted a protein of 140 amino acid residues and a deduced *M*_r_ = 15,836, with a signal peptide of 19 residues. Thus, the predicted mature polypeptide had a *M*_r_ = 14,137 and a sequence distinct from known cytokines, including IL-1, IL-2, IL-3, GM-CSF, and IFN_αβγ_. Biological assays indicated that IgG_1_-inducing activity, IgG_2/3_-reducing activity, B cell Ia-inducing activity, and BCGF-I activity were all attributable to the same protein. Accordingly, these investigators proposed that this activity be termed IL-4. An identical cDNA was isolated by Lee and coworkers in Ken-Ichi Arai’s group, who also showed that this new lymphokine had TCGF and mast cell growth factor activities, as well as IgE inducing activity (120).

Others working contemporaneously focused on TRF activity and BCGF-II activity, trying to understand exactly how many lymphokine molecules were involved in promoting B cell proliferation and differentiation. Like the evolution of IL-3, which eventually was found to have growth factor activity for multipotential hematopoietic cells, Sanderson and coworkers found that an eosinophil differentiating factor (EDF) also promoted the growth and differentiation of BCL_1_ lymphoma cells, thereby suggesting that a single might target both myeloid and lymphoid cells (121). Their evidence derived from a significant correlation between the EDF and BCGF-II assays when supernatants from 39 separate T cell clones were tested, as well as coincidence in gel filtration and HPLC elution profiles. Subsequently, Takatsu and Severinson collaborated with Honjo’s group, and identified a cDNA clone that coded for 133 amino acid residues containing a 21 residue leader sequence and a mature protein of *M*_r_ = 12,300 (122). The entire amino acid sequence was unique, but contained short sequences homologous to those of IL-3, GM-CSF, and IFN_γ_, thereby suggesting that it belonged to a cytokine family. Because this new and novel protein demonstrated both TRF and BCGF-II activities, it was proposed to be termed IL-5.

Independently of all of these efforts, but focused on the same goals, Kishimoto’s group developed bioassays for human B cell Ig production from B cell lymphomas as well as B blast cells isolated from Staphylococcal-A-activated human tonsil cells (123). As a source of T cell-derived factors, they established a human T leukemia cell line that spontaneously released an activity that promoted Ig production. Hirano identified proteins of 19 and 21 kDa (*M*_r_), which he purified to homogeneity using several preparative steps, assaying relative concentrations using the reciprocal of the dilution that yielded 50% maximal Ig production as in the TCGF bioassay (123). cDNA probes were constructed based on amino acid sequences of the purified proteins, and used to identify clones from a cDNA library from the leukemic T cells that encoded a novel protein of 212 amino acid residues with 28 hydrophobic residues in the N-terminus (124). Thus, the mature protein consisted of 184 amino acids and a calculated *M*_r_ = 20, 782. Of the known cytokine sequences, only human G-CSF showed significant homology. Tests of biological activity of the purified protein indicated that this new cytokine promoted Ig production by both normal and malignant human B cells, but did not promote B cell proliferation (125). Subsequently, this molecule became known as IL-6.

Thus, more than 15 years after the first descriptions of macrophage and T cell-derived activities found to “help” AFC formation and Ig production, three new interleukin molecules had been defined, isolated, characterized, and sequenced. Critical in this process were the development of new assays that were amenable to quantitative analysis like the IL-2 bioassay. However, despite this progress, none of the new interleukin molecules, either alone or in combination, could effectively promote long-term B cell proliferation and cloning as had been so successful using IL-2 to create T cell lines and clones. Accordingly, there seemed to be something missing, or different about activation and proliferation of B cells compared with T cells.

Looking back to “linked recognition” (95), the observation that T cells contributed some vital physical signal to B cells independently of soluble helper molecules had never been fully explained. Thus, a report from Clark and Ledbetter of a MoAb that recognized a 50 kDa (*M*_r_) surface molecule restricted to normal and neoplastic B cells was of interest, in that this MoAb augmented the proliferation of anti-IgM-activated human tonsil B cells (126). This B cell surface molecule was subsequently given a classification of CD40, and Blanchereau’s group then demonstrated the first long-term growth of human B cell lines using IL-4 and anti-CD40 (127). Because it had been shown that resting T cells proliferate to anti-CD3 bound to mouse fibroblastic cells expressing FcγRII, several MoAbs reactive with various B cell surface molecules were tested with these FcR+ cells for their capacity to activate the proliferation of highly purified resting human B cells. Only MoAbs reactive with CD40 induced proliferation. Particularly noteworthy, addition of recombinant IL-4 strongly enhanced the B cell proliferation, whereas the addition of IL-2 only weakly enhanced proliferation. B cells could be cultured with this combination for up to 10 weeks, and could be cloned by limiting dilution using the anti-CD40 MoAb bound to the FcγRII-expressing cells + IL-4.

Then within the span of a year, four groups reported finding a surface molecule expressed by mitogen-activated Th cells that bound to B cell CD40 and activated both their proliferation and differentiation to AFCs. Lederman working in Chess’s group found that a clone of the human T leukemia cell line JURKAT possessed the unique capacity to induce the expression of B cell CD23. Using this clone, a MoAb was generated that bound to the T cell clone and inhibited activated T cells from inducing B cell CD23 expression (128, 129). Similarly, Spriggs’ group found that human CD40 bound to the murine thymoma cell line, EL4. Using an expression cDNA library constructed from EL4 mRNA and biotinylated CD40, a cDNA was cloned that encoded a 260 amino acid residue protein with a calculated *M*_r_ = 29,395, which they termed CD40-ligand (CD40-L). cDNA transfected paraformaldehyde-fixed cells activated the proliferation of murine splenic B cells and the addition of IL-4 promoted their production of IgE. In addition, Noelle’s group had already reported that paraformaldehyde-fixed mitogen-activated Th cells and their purified plasma membranes induced B cell cycle entry and that IL-4, but not IL-2, IL-5, or IFN_γ_, augmented B cell DNA synthesis (130). Using a human CD40-Ig fusion protein, they identified a 39 kDa surface molecule on activated but not resting Th cells, and raised a MoAb that reacted with this protein that blocked the induction of B cell cycle entry (131). Finally, two separate groups isolated human cDNA clones encoding the human homolog of the murine CD40-L (132, 133).

The power of this molecular and genetic insight thus gained was immediately realized by reports from three groups and a total of 10 individuals who suffered from X-linked Hyper-IgM syndrome, which is a rare genetic disorder characterized by normal or elevated levels of serum IgM but undetectable levels of IgG, IgA, and IgE (134–136). Circulating B cell concentrations were normal, but the cells exclusively expressed only surface IgM/IgD. Affected males are unusually susceptible to recurrent pyogenic infections, autoimmune diseases, and lymphoproliferative diseases, thus a curious paradoxical mixture of immunodeficiency and autoimmunity, very similar to neonatal thymectomized mice (39). Genetic mapping of the CD40-L gene revealed that it is located in the q26.3-q27.1 region of the X chromosome (132), while X-linked Hyper-IgM syndrome had previously been assigned to Xq26. Various genetic mutations in the 10 individuals accounted for either the total lack of CD40-L expression, or to expression of CD40-L protein that failed to bind CD40. Accordingly, these reports solidified the only non-redundant function of the CD40-L/CD40 interaction is the important Ig class switch recombination that governs the switch from the μ-constant region to the other constant region H-chain isotypes.

## Conclusion

In the decade after the discovery of the molecular nature of the TCR complex and the realization that T cell effector molecules are antigen non-specific leukocytotrophic hormones, it was possible to begin to identify, isolate, and characterize the molecules responsible for adaptive immunity for the first time. Thus, the biochemical analysis of antigen-specific TCR signaling revealed that lymphocytes are excitable cells, not too different from neuromuscular cells that react to their molecular environment by signaling through their lipid membranes, leading to ion fluxes and activation of phosphorylating enzyme cascades that regulate the genetic expression of the cells. Among the first and most important of the molecules encoded by the genes activated by the TCR were the molecules involved in cytolysis and helper functions. The biochemical understanding of apoptosis provided a molecular process by which negative selection of immature lymphocytes reactive with self-molecules provided a firm molecular underpinning of Burnet’s prediction that self-reactive clones would be eliminated during development. T cell clones and cloned TCR genes permitted the generation transgenic animals that were instrumental in demonstrating that the phenomena of negative and positive selection actually occur. The molecular explanation of T cell help of B cell proliferation and differentiation to AFCs, as residing in both soluble interleukin molecules as well as cell surface ligand/receptor pairs, yielded a new understanding of how messages are passed between the cells comprising the immune system. The unraveling of the various new interleukin molecules involved in T cell help was long and laborious but was ultimately successful because of the use of cloned T cells and the creation of quantitative bioassays, amenable to biochemical and genetic analysis of the various activities. The initial results of these advances immediately became apparent in new understanding of genetic mutations responsible for primary immunodeficiencies. Thus, the pathways were opened to a further molecular delineation, dissection, and elucidation of the TCR complex, the various interleukin receptors, the biochemical signaling pathways, and genes activated by these newly discovered adaptive immune molecules.

## Conflict of Interest Statement

The author declares that the research was conducted in the absence of any commercial or financial relationships that could be construed as a potential conflict of interest.
